# Research on Adaptive Machining Technology for Aluminum Alloy Free-Form Surfaces

**DOI:** 10.3390/ma19153312

**Published:** 2026-08-04

**Authors:** Wenxia Zhang, Yangjun Wang

**Affiliations:** School of Mechanical and Electrical Engineering, Soochow University, Suzhou 215000, China; wenxzhang12@163.com

**Keywords:** line laser scanning, visual positioning, point cloud processing, trajectory planning, NURBS surface, CNC milling

## Abstract

In conventional CNC machining, the workpiece clamping pose is registered with a preset CAD model under multiple geometric constraints to establish the machining reference frame. The tool path, generated from this model, is subsequently used to produce components of identical geometry. However, this paradigm proves inadequate when a final shape must accommodate morphological variations specific to each individual blank. Manual grinding, as an alternative, is not only inefficient and hazardous but also relies heavily on subjective quality assessment. To address these challenges, we propose an adaptive local-region milling strategy tailored for blanks with similar yet non-identical surface morphologies, enabling the finished geometry to adjust dynamically to each workpiece. Under conditions of under-constrained clamping, visual positioning is first employed to automatically locate the target regions. Line laser scanning is then conducted over the planned area to acquire high-density point clouds. Through segmentation, points lying outside the region to be machined are extracted, from which a theoretical post-machining surface is reconstructed. Milling toolpaths are subsequently planned based on this reconstructed model to compensate for surface variations across different blanks. Experimental validation on a three-axis CNC milling machine demonstrates that the proposed adaptive strategy effectively replaces manual grinding by removing the bulk of the machining allowance from locally variant surfaces. With the reconstructed model serving as the reference, 77.1 percent of the machining errors fall below 0.055 mm. These results confirm that the method yields a smooth and level surface finish, thereby meeting the fundamental requirements for such adaptive machining tasks.

## 1. Introduction

Curved components, such as precision dies and aero-engine blades [1,2,3,4,5], are extensively employed in the automotive and aerospace sectors. Their fabrication presents significant challenges, including uneven machining allowances that induce abrupt cutting-force fluctuations, low efficiency and accuracy in manual alignment, and considerable difficulties in the fine positioning of large or heavy workpieces, which in turn hinders precise fixturing on machine tools [6,7]. Adaptive machining technology mitigates these issues through the deployment of diverse sensing modalities for real-time monitoring and feedback, thereby enabling automatic adjustment and optimization of machining parameters to enhance both efficiency and quality. Geometric adaptation, in particular, adjusts tool paths in response to the actual shape or position of the part, thereby compensating for clamping inaccuracies, workpiece deformation, or non-uniform allowances [8]. For instance, Autodesk’s Adaptive Machining compares measured data—acquired via on-machine inspection or coordinate measuring machines—against the CAD model and modifies the tool paths accordingly.

Three-dimensional measurement is a critical enabler of geometric adaptive machining, as it allows for the rapid acquisition of surface morphology, thereby furnishing essential data for process strategy formulation. Non-contact measurement methods, characterized by their stability, efficiency, and non-destructive nature, are extensively adopted in this context [9]. Several prior studies have demonstrated specific applications: Liu et al. [10] combined a line laser rangefinder with a displacement sensor for on-machine measurement of large shafts, relying on spatial analytic geometry; Xu et al. [11] integrated a line laser scanner into a robotic laser-aided additive manufacturing system for in situ geometry measurement and proposed an adaptive size-correction strategy to mitigate cumulative deviation-induced failures; and Seo et al. [12] employed LiDAR to acquire point cloud data of target structures and coupled it with the particle difference method to evaluate deformation and stress [13]. Among these non-contact techniques, line laser scanning offers a broader scan range than point laser sensors [14] while delivering superior accuracy and acquisition frequency compared to LiDAR, particularly when integrated with mechanical positioning systems. These advantages render it a widely adopted solution for on-machine measurement in manufacturing.

Despite these advances, nearly all existing adaptive machining strategies are predicated on correcting deviations from a fixed CAD model to produce geometrically identical parts [15]. This underlying assumption becomes invalid when the final shape must be tailored to the unique morphology of each raw blank—a common scenario in customized production, wherein current practice still relies on manual grinding or polishing. Such manual operations are particularly problematic for aluminum alloys, as they generate hazardous dust [16,17]. Under a fixed CAD paradigm, milling is rendered infeasible by the unpredictable geometry of the blanks and the absence of a nominal target surface.

To address this limitation, we propose a blank-conforming adaptive machining paradigm that derives tool paths directly from measured blank geometry, circumventing reliance on a nominal CAD model. Our framework redefines the adaptation objective and integrates calibration, localization, surface reconstruction, and path planning within a unified closed-loop system that operates independently of pre-existing geometry. In contrast to these existing strategies that rely on a nominal CAD model for deviation correction, the proposed method operates without a pre-existing geometric reference. For instance, the adaptive milling approach reported by Cui et al. for aero-engine blades still requires a deformation mapping relationship derived from the CAD model to modify the tool path. Similarly, the robotic edge milling method developed by Xu et al. for large curved thin-walled parts depends on measured data but ultimately corrects deviations back to a nominal design surface. The robotic laser cladding system presented by Ma et al. for freeform surface remanufacturing employs a line laser scanner for geometry acquisition, yet the trajectory planning remains referenced to the nominal model of the repair target. Among the reviewed studies, only a limited number have addressed the scenario where the final surface must conform to the specific morphology of each individual blank, and these typically resort to manual grinding or polishing operations due to the absence of a feasible automated milling solution. The proposed framework distinguishes itself by integrating vision-based self-localization, line laser scanning, on-machine surface reconstruction, and automated tool path generation within a unified closed-loop system. This configuration not only eliminates the reliance on a fixed CAD model but also achieves a machining accuracy with 77.1 percent of errors below 0.055 mm and a maximum error below 0.1 mm, which is competitive with or superior to the reported accuracies of existing CAD-referenced adaptive machining approaches. Furthermore, the total processing time of approximately 6 min per workpiece and the ability to handle geometrically varying blanks make it more efficient and consistent than manual finishing [18,19,20]. By synergizing vision-based self-localization with line laser scanning, our method enables dynamic path adjustment for non-uniform workpieces, thereby substantially enhancing both flexibility and operational safety.

## 2. Adaptive Processing System Structure and Calibration

### 2.1. Adaptive Processing System Structure

**Figure 1:**
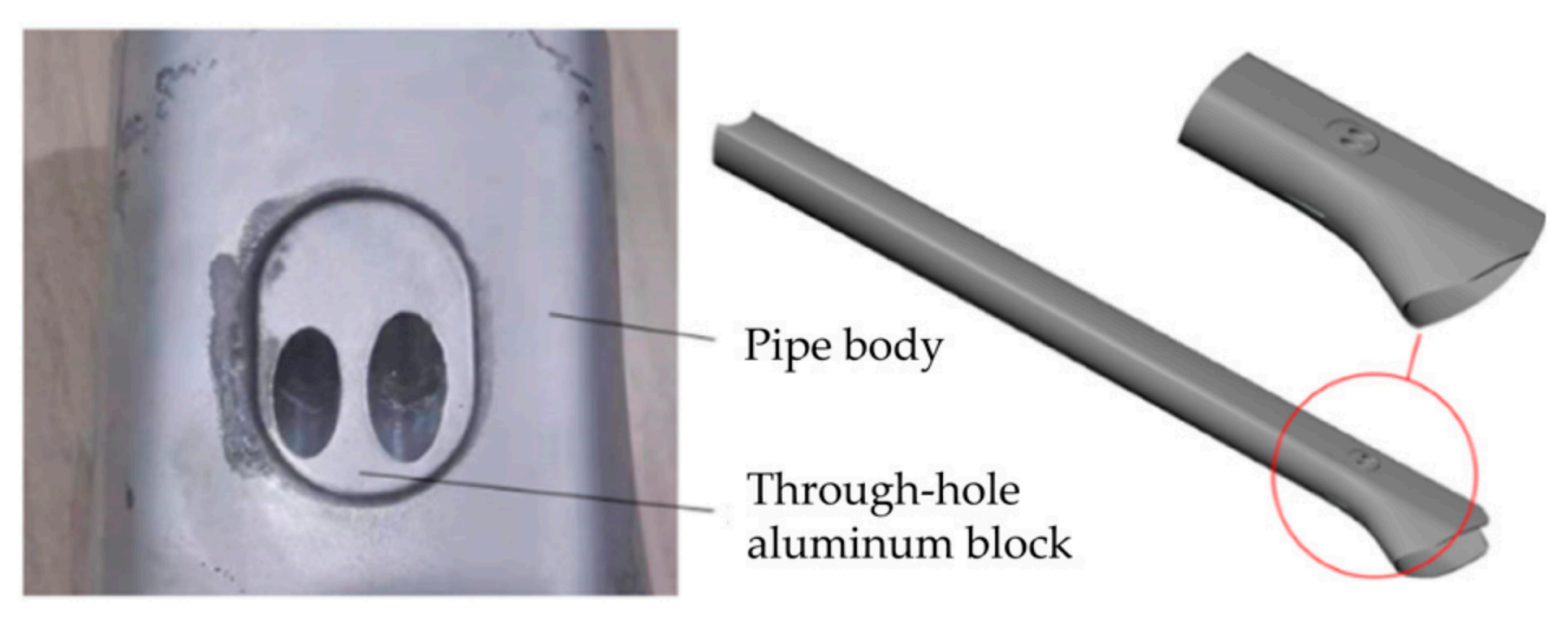
Vehicle tube workpiece.

The component illustrated in Figure 1 is a critical part in bicycle frame manufacturing, with an annual production volume reaching millions of units. It is fabricated by welding irregularly shaped aluminum alloy tubes to aluminum blocks that contain through-holes, followed by a punching operation. This manufacturing process inevitably generates local surface protrusions in the vicinity of the through-hole blocks. Since these protrusions adversely affect the subsequent painting process, they must be removed to achieve a smooth and flush surface finish on the tube. However, the punching operation introduces irregular deformations on the tube surface and causes significant positional variations in the protrusions across different blanks. Consequently, planning the machining trajectory based on a unified nominal model becomes infeasible.

This study focuses on the aforementioned component and proposes a geometric adaptive milling strategy that integrates visual positioning with line laser scanning. The experimental system comprises a line laser scanner with its controller, an industrial camera, a CNC machine tool, and an industrial control unit.

The workflow is as follows:

1. The outer surface of the bicycle frame tube is an irregular free-form surface, so a conformal fixture is used to clamp the workpiece, with the target face kept as horizontal as possible;
2. The CNC spindle moves to a fixed camera position above the fixture to capture images of the processing area and its surroundings. Template matching yields the image coordinates of the area, and calibration between the camera and workpiece coordinate systems gives the workpiece system coordinates, which serve as the reference for line laser scanning;
3. Based on the position from step 2, the scanning area and path of the line laser scanner are planned. The square-wave pulses generated by the grating ruler and reading head trigger the acquisition of 3D point clouds of the local surface. Stitching and filtering (denoising and smoothing) are applied if necessary;
4. From the obtained point clouds, the outer shell of the tube and the closed boundary curve of the through-hole aluminum block are extracted as the boundary for trajectory planning. Surface fitting reconstructs the machining surface model, from which a milling trajectory is generated. The machining is then carried out to smooth and flatten the target area.

### 2.2. Calibration of the On-Machine Measurement System

The kinematic model of the geometric adaptive processing system described in this article is established based on a three-axis CNC machine. The machine measurement system includes the workpiece coordinate system, the camera coordinate system, the scanner coordinate system, and the tool coordinate system. Among them, the line laser measuring instrument adopts the principle of laser triangulation. *Z_S_* and *X_S_* represent the direction of laser emission and the direction of line length, respectively. When the measuring instrument and the measured object have relative motion, a coordinate system *Y_S_* axis is generated.

(1) PWa=PWT+TST·PaS,

Among them, TST represents the transformation relationship between the scanner coordinate system and the tool coordinate system, which is the hand–eye matrix. PWT is the transformation matrix for converting the tool coordinate system to the workpiece coordinate system, which is read by the machine tool numerical control system. PSa is the measurement point in the line laser scanner coordinate system, and PWa is the position of the specified measurement point in the workpiece coordinate system.

(2) TST=RSTS0T1, PWa1=T01,

Equation (1) can be transformed into Equation (3).

(3) PWa1=T01+RSTS0T1×PSa1,

Since a three-axis CNC machine tool is used to construct the processing system, after installing the line scanner at a fixed position, there is only relative translational motion between the scanner and the scanning object. Then, the following method is adopted to solve the rotation matrix $R_{S}$ and the translation matrix $T_{S}$ in the eye–point matrix step by step [21]:

1. Solving the rotation matrix $R_{S}$

Multiple scans are conducted on the standard ceramic ball fixed within the processing area of the CNC machine tool. The rotation matrix $R_{S}$ is solved using the consistency of the ball center.

Let point $P_{S}$ in the scanner coordinate system correspond to point $P_{t}$ in the tool coordinate system. Then, $P_{t}\;=R_{S}\cdot P_{S}\;+T_{S}\;+T_{0}$. By controlling the line scanner to scan the standard ball at two different positions, the following can be obtained:

(4) $$\left\{\begin{array}{c} P_{t}\;=R_{S}\cdot P_{S1}+T_{S}+T_{0_{1}}\\ P_{t}\;=R_{S}\cdot P_{S2}+T_{S}+T_{0_{2}} \end{array}\right.,$$

By subtracting the two equations, we get $R_{S}\cdot {(P}_{S1}-P_{S2})=T_{0_{2}}-T_{0_{1}}$. Multiple measurements are carried out on different parts of the ceramic balls and the following solution equation is established:

(5) $$R_{S}\cdot [P_{S1}-P_{S2}\;P_{S1}-P_{S3}\;P_{S1}-P_{S4}{\;P}_{S1}-P_{S5}]={[T}_{0_{2}}-T_{0_{1}}\;T_{0_{3}}-T_{0_{1}}\;T_{0_{4}}-T_{0_{1}}\;T_{0_{5}}-T_{0_{1}}],$$

Then, the rotation matrix $R_{S}$ can be obtained by solving the least squares solution of this equation.

Based on the relative positional relationship between the laser plane and the standard sphere, the process for determining the coordinate position of the sphere center $P_{S}(x_{s},y_{s},z_{s})$ in the laser scanner is as follows: Using the least squares method to fit the position of a single frame of scanning, the intersection point $O(x_{o},y_{o})$ of the light plane of the scanner and the ceramic standard sphere in the plane is the center coordinate point of the circle. Then, the position $P_{S}\left(x_{s},y_{s},z_{s}\right)$ of the sphere center in the scanner coordinate system is:

(6) $$\left\{\begin{array}{c} x_{s}\;=\;x_{o}\\ y_{s}\text{±}\sqrt{R^{2}-r^{2}}\\ z_{s=}y_{o} \end{array}\right.,$$

R represents the radius of the standard sphere, and r represents the radius of the fitted circle. The sign of $y_{s}$ needs to be determined based on the part of the standard sphere scanned by the line laser scanner. After obtaining the center coordinates of the sphere at each scanning position, the rotation matrix $R_{S}$ is calculated according to Equation (5).

2. Solving the translation matrix $T_{S}$

After obtaining the rotation matrix, the unknown parameters in the hand–eye matrix are reduced to 3 elements in the translation matrix $T_{S}$, namely $T_{S}\;={\left(x_{\Delta },y_{\Delta },z_{\Delta }\right)}^{T}$.

(7) $$\left[\begin{array}{c} x_{w}\\ y_{w}\\ z_{w}\\ 1 \end{array}\right]=\left[\begin{array}{cccc} r_{11} & r_{12} & r_{13} & x_{\Delta }\\ r_{21} & r_{22} & r_{23} & y_{\Delta }\\ r_{31} & r_{32} & r_{33} & z_{\Delta }\\ 0 & 0 & 0 & 1 \end{array}\right]\left[\begin{array}{c} x_{s}\\ y_{s}\\ z_{s}\\ 1 \end{array}\right]+\left[\begin{array}{c} x_{0}\\ y_{0}\\ z_{0}\\ 1 \end{array}\right],$$

where $\left(x_{w},y_{w},z_{w}\right)$ are the workpiece coordinates. From the above equation, we can conclude:

(8) $$\left\{\begin{array}{c} x_{\Delta }=x_{W}-(r_{11}\cdot x_{s}+r_{12}\cdot y_{s}+r_{13}\cdot z_{s}+x_{0})\\ y_{\Delta }=y_{W}\;-(r_{21}\cdot x_{s}+r_{22}\cdot y_{s}+r_{23}\cdot z_{s}+y_{0})\;\\ z_{\Delta }=z_{W}\;-(r_{31}\cdot x_{s}+r_{32}\cdot y_{s}+r_{33}\cdot z_{s}+z_{0}) \end{array}\right.,$$

**Figure 2:**
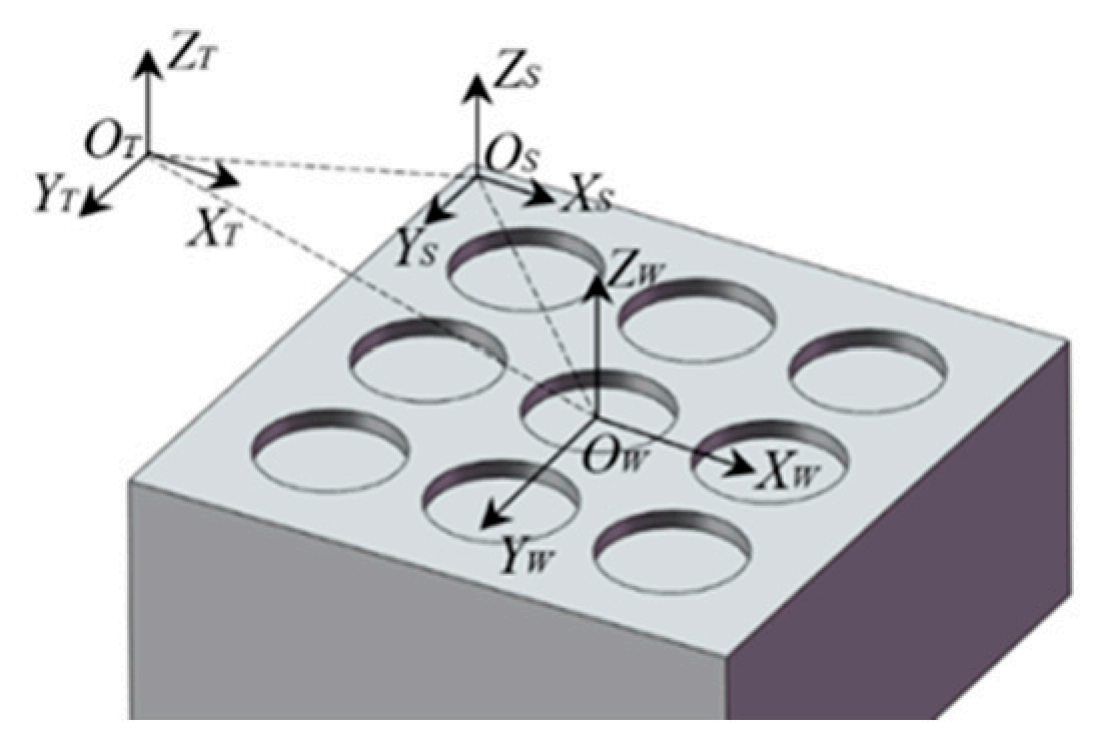
The translation matrix diagram is solved by calibration block.

From Equation (8), it can be seen that when the rotation matrix is known, the corresponding relationship between a point in the space and the scanner coordinate system and the tool coordinate system can be obtained, thereby solving the translation matrix. Therefore, in the CNC machine tool, the circular slot array calibration block is processed as shown in Figure 2. The upper surface of the calibration block is the $Z_{W}\;=\;0$ reference plane of the workpiece coordinate system. The circular centers of the nine circular slots in this reference plane are used as the marking points. The workpiece coordinates of the marking points are taken at the theoretical position, that is, $\left(x_{si},y_{si},z_{si}\right)$. The complete point cloud information of the circular slot array is obtained, and based on the scanning starting point position, the coordinate positions $\left(x_{si},y_{si},z_{si}\right)$ of the centers of each circular contour in the point cloud are detected. Equation (8) is used to take the average of the nine calculation results, which is the translation matrix $T_{S}={\left(x_{\Delta },y_{\Delta },z_{\Delta }\right)}^{T}$.

Using the above calibration method, the calibration results of the line laser scanner and the hand–eye matrix of the tool coordinate system in this paper are as follows. In practical applications, when converting the point cloud coordinates to the tool coordinate system, the calculation should be carried out based on the starting coordinates of each scan position and combined with the hand–eye matrix.

(9) TST=0.9998230.018749−0.001793−135.713−0.0187490.9998240.000364 64.2170.0018000−0.00033000.999998−7.66001,

The industrial camera uses DH-HV3151UC-M from Haodian Image (IMAVISION, Beijing, China), and the lens is HN-P-0824-10M-C2/3, with a pixel size of 3.2 μm. The visual system is calibrated with the nine-point calibration method between the numerical control machine tool and the coordinate system *O_M_X_M_Y_M_* of the workpiece. The coordinate transformation relationship between the imaging plane of the industrial camera and the coordinate plane *O_M_X_M_Y_M_* of the workpiece is calculated. The relationship between the camera coordinates $\left(x_{c},y_{c}\right)$ and the workpiece coordinates ${(x}_{w},y_{w})$ is as follows:

(10) $$\left[\begin{array}{c} x_{w}\\ y_{w}\\ 1 \end{array}\right]=\left[\begin{array}{cc} R & T\\ O^{T} & 1 \end{array}\right]\left[\begin{array}{c} x_{c}\\ y_{c}\\ 1 \end{array}\right]=\left[\begin{array}{ccc} r_{11} & r_{12} & r_{13}\\ r_{21} & r_{22} & r_{23}\\ 0 & 0 & 1 \end{array}\right]\left[\begin{array}{c} x_{c}\\ y_{c}\\ 1 \end{array}\right],$$

By capturing the image coordinates of nine marker points and their corresponding workpiece coordinates, the image coordinates of the nine markers are $\left(x_{\mathrm{ci}},y_{\mathrm{ci}}\right)\left(i\;=1,2,3\ldots \right)$, and their corresponding positions in the workpiece coordinate system are $(x_{\mathrm{wi}},y_{\mathrm{wi}})\left(i\;=1,2,3\ldots \right)$. Then, based on the least squares solution of the equation AX =Y , given by $\hat{X}={\left(A^{T}A\right)}^{-1}A^{T}Y$, the least squares solution is computed. Let:

(11) $$A=\;{\left({\left[\begin{array}{ccc} x_{c1} & y_{c1} & 1\\ \vdots & \vdots & \vdots \\ x_{c9} & y_{c9} & 1 \end{array}\right]}^{T}\left[\begin{array}{ccc} x_{c1} & y_{c1} & 1\\ \vdots & \vdots & \vdots \\ x_{c9} & y_{c9} & 1 \end{array}\right]\right)}^{-1}{\left[\begin{array}{ccc} x_{c1} & y_{c1} & 1\\ \vdots & \vdots & \vdots \\ x_{c9} & y_{c9} & 1 \end{array}\right]}^{T},$$

then,

(12) $$\left[\begin{array}{c} r_{11}\\ r_{12}\\ r_{13} \end{array}\right]=A\left[\begin{array}{c} x_{m1}\\ \vdots \\ x_{m9} \end{array}\right],\left[\begin{array}{c} r_{21}\\ r_{22}\\ r_{23} \end{array}\right]=A\left[\begin{array}{c} y_{m1}\\ \vdots \\ y_{m9} \end{array}\right],$$

After determining the shooting position, when solving the position of the area to be processed, the parameters of the extrinsic matrix are constants. The calibration results of the industrial camera are:

(13) $$\left[\begin{array}{cc} R & T\\ O^{T} & 1 \end{array}\right]=\left[\begin{array}{ccc} -0.000635 & -0.057946 & 61.023\\ 0.057983 & -0.000597 & -49.422\\ 0 & 0 & 1 \end{array}\right],$$

By applying this calibration result, the position of the area to be processed in the camera coordinate system can be converted to the machine coordinate system, enabling the preliminary positioning of the processing area. This can be used as a reference position for planning the line scanning area.

## 3. Autonomous Positioning of Processing Area and Point Cloud Acquisition

### 3.1. Autonomous Positioning of Processing Area

Due to the randomness of the positions of the areas that need to be processed on the workpiece surface, and the under-constrained clamping of the workpiece in the CNC machine, the pose of the processing area in the machine coordinate system is uncertain. At the same time, due to the limitation of the scanning range of the line laser scanner, the processing area needs to be preliminarily positioned before online scanning to plan an appropriate scanning path. By using the gray feature of the processing area, the normalized cross-correlation coefficient method is adopted for template matching to complete the autonomous positioning of the processing area [22]. The principle of the algorithm is to calculate the correlation coefficient between the real-time image and the reference image using the correlation coefficient formula as the similarity measurement function, and obtain the correlation coefficient matrix, defined as ncc(r,c):

(14) $$\frac{1}{n}\sum_{(u,v)\in R}\frac{t(u,v)-m_{t}}{\sqrt{s_{t}^{2}}}\cdot \frac{i(r+u,c+v)-m_{i}(r,c)}{\sqrt{s_{t}^{2}(r,c)}},$$

Here, n represents the number of points in the template, and R represents the domain of the template.

The average gray value of the template is:

(15) $$m_{t}=\frac{1}{n}\sum_{(u,v)\in R}t(u,v),$$

The variance in the template’s gray value is:

(16) $$s_{t}^{2}=\frac{1}{n}{\sum_{(u,v)\in R}\left(t(u,v)-m_{t}\right)}^{2},$$

The average gray value of the image region overlapped by the template at position (r,c) is.

(17) $$m_{i}(r,c)=\frac{1}{n}\sum_{(u,v)\in R}i(r\;+u,c\;+v),$$

The variance of the gray values of the image region overlapped by the template at position (r,c) is:

(18) $$s_{i}^{2}(r,c)=\frac{1}{n}{\sum_{(u,v)\in R}\left(i(r+u,c+v)-m_{i}(r,c)\right)}^{2},$$

**Figure 3:**
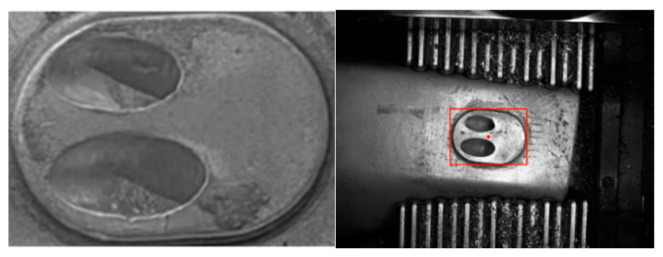
Template image and matching results block.The red dot in the right image indicates the centroid of the matched region.

The matching is performed using the template image of the processing area, as shown in Figure 3. The position with the highest similarity is taken as the matching result. The feedback result is the image coordinate of the center position of the area in the image that is most similar to the template, which indicates the approximate position of the center of the processing area in the image.

The result of the template matching, that is, the center position of the processing area in the image, is converted to the coordinate system of the workpiece. Using this position, the scanning area is planned, and the line scanning path is quickly obtained. The point cloud data of the measured surface is accurately acquired, avoiding invalid travel.

### 3.2. Acquisition of Point Cloud Data for Scan Path Planning

To obtain sufficient point cloud data, a theoretical processing effect surface model needs to be constructed. This requires obtaining the surface point cloud information around the area to be processed. For this purpose, a rectangular line laser scanning area of a fixed size is planned. This rectangular area is centered on the midpoint coordinates of the area obtained through template matching. The length and width of this rectangular area are approximately 1.5 times that of the area to be processed, covering the through-hole aluminum block and the surrounding pipe body surface area.

**Figure 4:**
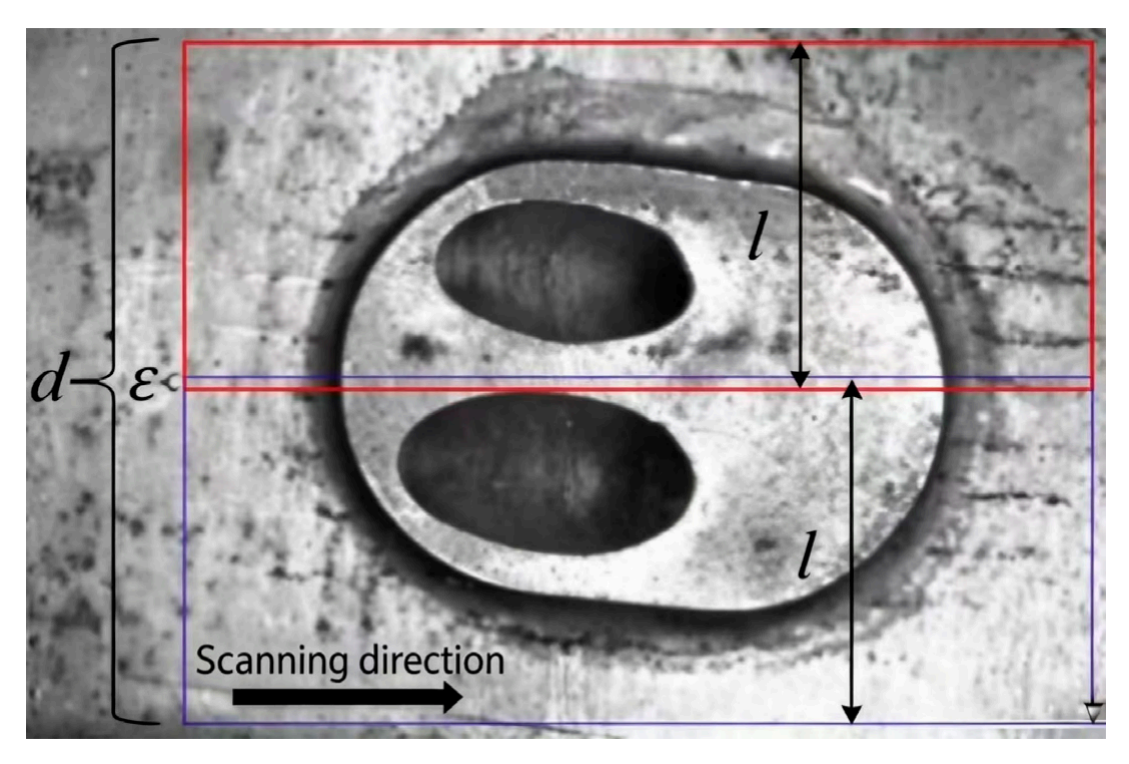
Scan area division diagram. The red and blue squares represent two separate scanning regions, respectively.

The scan path is planned based on this area, as shown in Figure 4. Let the line width of the selected line laser profile measurement instrument be l, and the width of the area to be scanned be d, satisfying d=2l−ε, where ε is the width of the repeated scanning area for two scans, used to correct the stitching error in the depth direction. After the first part of the scan is completed, the spindle carrying the line laser scanner moves a distance of l−ε in the length direction. Starting from the same starting position as the first scan direction, the scan of the other part of the planned area is completed. Due to the limited clamping height of the workpiece, the height variation range of the area to be processed in the fixture is small. In contrast, the scanning depth range of the scanner is larger, so the scan is always performed at a fixed scanning height.

The point cloud information of the area is obtained by using the above scanning strategy. However, due to the scanning depth limitation of the line laser scanner, some invalid areas exceed the scanning range, resulting in redundant points. Alternatively, due to the complex surface condition of the workpiece, noise point clouds may be generated. After obtaining the original point cloud information through two scans, direct filtering is used to divide and retain the points within the effective measurement range of the point cloud, and then radius filtering [23] is used to remove the discrete noise points. The specific method is as follows:

1. The workpiece is under-located and its spatial position cannot be determined, but the spatial area that can enclose the valid point cloud data has a certain range. After statistical analysis, a relatively appropriate scanning area point cloud retention range of 45 mm long and 31.5 mm wide is set;
2. Calculate the centroid of the single-sided scanning point cloud based on Equation (19). Taking the centroid point $P_{c}$ as the center, retain the point cloud within the set size range, thereby removing most of the redundant points;
  (19) $$P_{c}=\frac{1}{n}\left(\sum_{i=0}^{n}x_{i},\sum_{i=0}^{n}y_{i},\sum_{i=0}^{n}z_{i}\right),$$
3. Use radius filtering to remove outliers. Set a sphere neighborhood with a radius of r and a quantity threshold of $N_{threshold}$ Traverse each point. If the number of points within the neighborhood of a certain point is less than $N_{threshold}$, consider that point an outlier and remove it.

**Figure 5:**
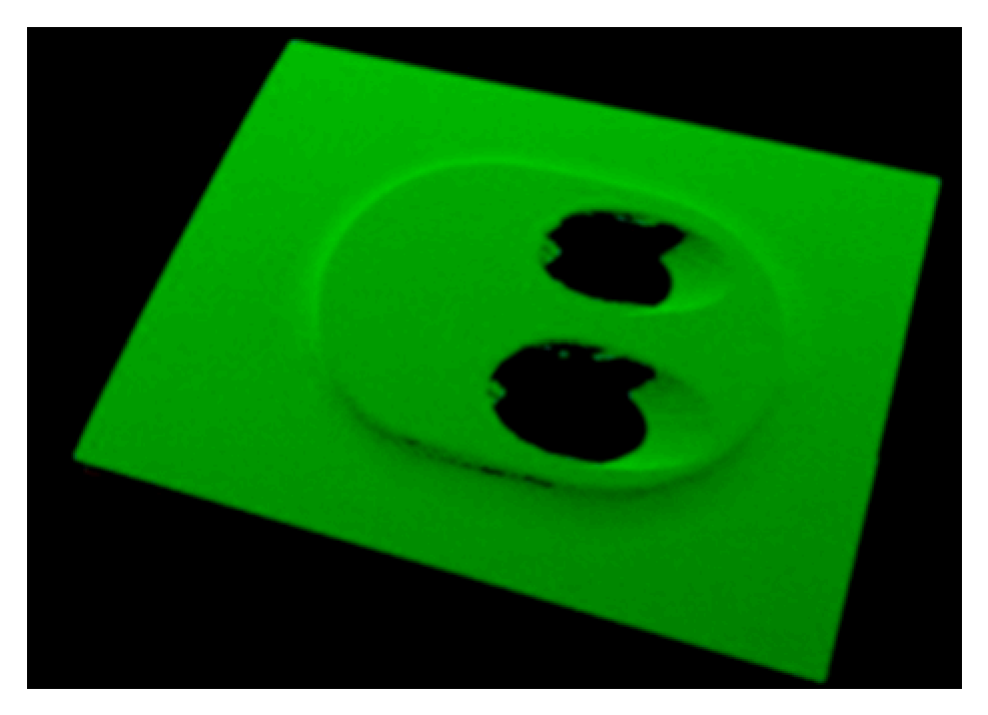
Scan area complete point cloud.

The processed point cloud is concatenated and fused by using the scanning point cloud method. The complete processed point cloud is shown in Figure 5. Thus, the positioning and precise measurement of the processing area have been completed. The obtained point cloud data can be downsampled to improve the efficiency of subsequent calculations.

## 4. Processing Area Boundary Extraction and Surface Reconstruction

The pivotal step in constructing the theoretical post-machining surface model is the extraction of the point cloud corresponding to the tube shell region, which serves as the fitting data. Leveraging the curvature variation characteristics at the boundary of the through-hole aluminum block, a region-growing algorithm based on curvature and normal vectors is employed to segment the tube shell point cloud.

In this study, after establishing the point cloud topological structure using a k-d tree, region-growing segmentation is performed on the scattered point cloud data. The point with the minimum curvature is selected as the initial seed point to initiate the growing process. The affiliation of a neighboring point to the current surface is determined by evaluating both the normal-vector difference and the curvature relationship between the seed point and its neighbors. Specifically, the angular deviation between the normal angle of each neighboring point and that of the current seed point is first computed; points with an angle below a preset threshold are incorporated into the current region. Subsequently, the curvature values of these neighboring points are examined, and those with curvature below the threshold are added to the seed point sequence for further growth. The algorithm terminates when the seed sequence becomes empty and the number of points in the segmented region exceeds the predefined minimum point count threshold [24].

**Figure 6:**
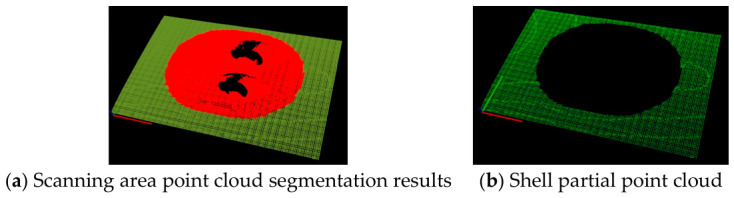
Point cloud results. The red points indicate the point clouds to be discarded, while the green points represent the retained point clouds of the tube shell.

The result of the area segmentation is shown in Figure 6a. The point cloud of the retained tube shell part is shown in Figure 6b.

To minimize non-productive milling paths and enhance machining efficiency, the toolpath region is confined exclusively to the through-hole aluminum block area. Accordingly, the closed curve fitted from the boundary point cloud of this region is employed as the constraint boundary, thereby restricting the machining trajectory to the designated zone.

To obtain the boundary of the processing area, the inner circle boundary of the point cloud of the tube shell part needs to be extracted first. This step is achieved by using the method of normal estimation to detect and extract the boundary, and using the angle criterion to estimate whether a group of points is on the surface boundary. The specific process is as follows: First, the point cloud data is organized using the k-d tree, and the normal vectors of each point are calculated; then, the angle $\alpha_{i}$ between the normal vector of each point and its *k* nearest neighbor points is counted successively, and the corresponding angle data set $A=\left\{\alpha_{1},\alpha_{2},\alpha_{3}\ldots \alpha_{n}\right\}$ is obtained. The maximum value of the angle data set is compared with the set threshold. If there is a maximum angle greater than the threshold, then this point is judged as the boundary point [25].

**Figure 7:**
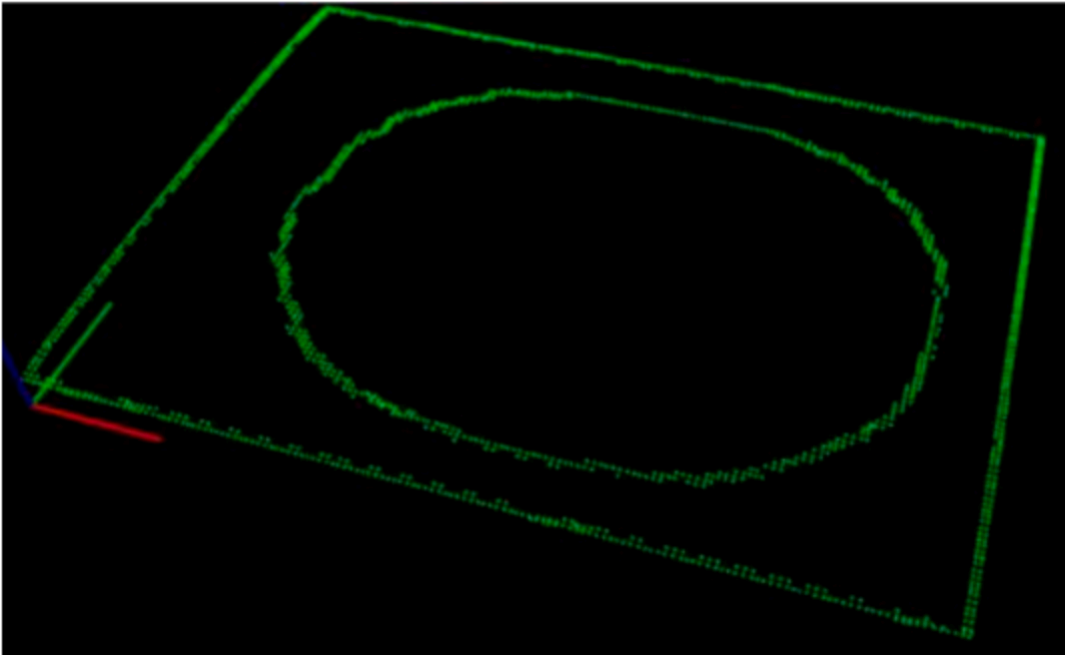
Boundary point cloud of shell part.

The point cloud boundary of the tube shell part extracted by this method is shown in Figure 7. The outer boundary part of the boundary point cloud is removed by the direct-through filtering method, and the internal boundary point cloud is downsampled to reduce the number of internal point clouds for fitting the boundary of the processing area and improve the fitting efficiency.

This internal boundary point cloud belongs to the point cloud of the shell part. By using an appropriate method, the curve fitted by this part of the point cloud can cover all the areas that need to be machined. In this paper, the projection points of the internal boundary point cloud on the o−xy plane are fitted using a cubic non-uniform closed B-spline curve, and the boundary of the area to be machined is constructed. When planning the machining trajectory, the x and y coordinates of the tool contact point are restricted within the fitted area [26].

Let $N_{i,k}(u)(i=1,2\ldots n)$ be the basis function of the kth B-spline, and the control vertices be $p_{i}(i=0,1,2\ldots n)$. The basis function adopts the De Boor–Cox recursive formula, and the expression of the kth B-spline is:

(20) $$p\left(u\right)=\sum_{i=0}^{n}p_{i}N_{i,k}\left(u\right),$$

After obtaining the point cloud of the boundary of the area to be processed and the boundary curve obtained through fitting, in order to simplify the subsequent calculation steps, equal parameter intervals are used to select boundary points on the boundary curve obtained through fitting, and each point is sequentially connected to form a closed line segment. This line segment combination is used to replace the B-spline curve of the boundary. Thus, the extraction of the milling trajectory boundary is completed, preparing for the subsequent trajectory planning.

## 5. Processing Effect Surface Establishment and Tool Path Planning

### 5.1. Theoretical Establishment of Processing Effect Surface

The processing effect surface model is constructed using NURBS surfaces [27]. A surface with p degrees of freedom in the u direction and q degrees of freedom in the v direction is set. Its analytical expression is:

(21) $$S\left(u,v\right)=\frac{\sum_{i=0}^{n}\sum_{j=0}^{m}N_{i,p}\left(u\right)N_{j,q}\left(v\right)w_{i,j}P_{i,j}}{\sum_{i=0}^{n}\sum_{j=0}^{m}N_{i,p}\left(u\right)N_{j,q}\left(v\right)w_{i,j}},0\leq u,v\leq 1,$$

**Figure 8:**
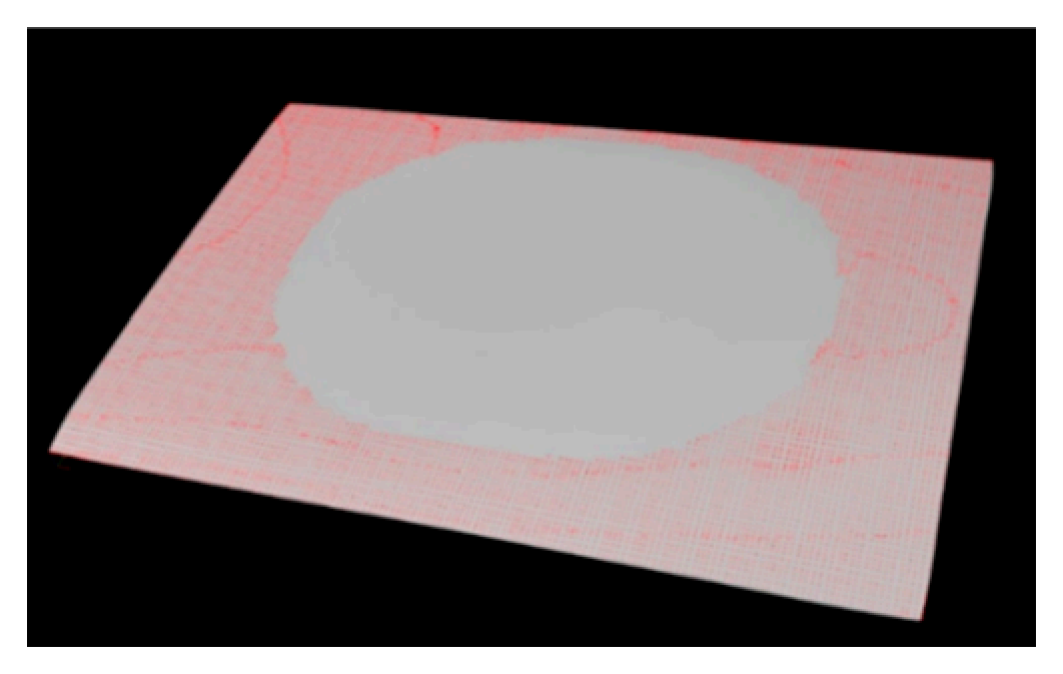
Theoretical machining effect surface mode (red points).

In this formula, $\left\{P_{i,j}\right\}$ forms the control grids in the u,v directions, $w_{i,j}$ is the weight factor for the control vertices, and $N_{i,p}\left(u\right)$ and ${\;N}_{i,q}\left(v\right)$ are the non-rational B-spline basis functions defined on the node vectors u and v. The NURBS surface established using the point cloud data of the shell part is shown in Figure 8. This surface model is the theoretical processing effect surface model. The calculation of the tool contact points and the planning of the milling trajectory are carried out on this surface.

### 5.2. Tool Path Planning

This paper adopts the use of ball-end milling cutters in a three-axis CNC machine for milling. During the processing, the tool axis is along the vertical direction, and milling needs to be performed from the normal direction. Let the surface unit normal vector at the tool contact point $A(x_{i},y_{i},z_{i})$ be $\vec{n_{i}}(n_{xi},n_{yi},n_{zi})$. Moving the tool contact point along this normal vector by a distance equal to the radius r of the ball-end milling cutter is the tool position point O. That is:

(22) $$O=\left[\begin{array}{c} x_{i}\\ y_{i}\\ z_{i} \end{array}\right]+r\times \left[\begin{array}{c} n_{xi}\\ n_{yi}\\ n_{zi} \end{array}\right],$$

For the NURBS surface $S\left(u,v\right)$, the partial derivatives with respect to parameters u and v at a certain point respectively yield the u-direction tangent vector and the v-direction tangent vector of the surface. Since the tangent vectors in the two directions are not collinear, as shown in Equation (23), the cross product of these two tangent vectors can be used to obtain the unit normal vector $A\left(u_{0},v_{0}\right)$ at point $n\left(u_{0},v_{0}\right)$ [28]. Using Equation (22), the tool position point *O* corresponding to the tool contact point *A* can be calculated.

(23) $$n\left(u_{0},v_{0}\right)=\frac{S_{u}\left(u_{0},v_{0}\right)\times S_{v}\left(u_{0},v_{0}\right)}{\left|S_{u}\left(u_{0},v_{0}\right)\times S_{v}\left(u_{0},v_{0}\right)\right|},$$

**Figure 9:**
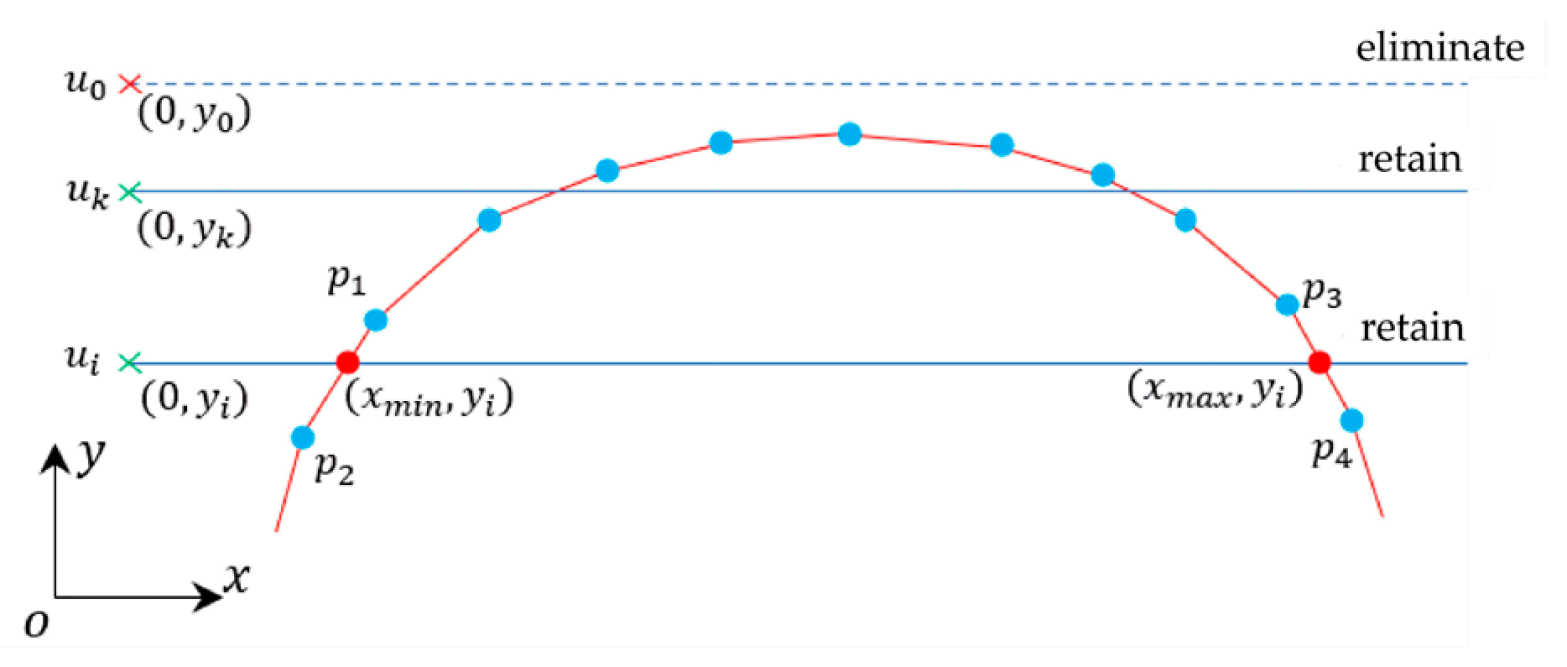
Schematic diagram of intersection of U-direction curve and calculation boundary (red curve: generated boundary curve; blue points: point sequence on the boundary curve).

**Figure 10:**
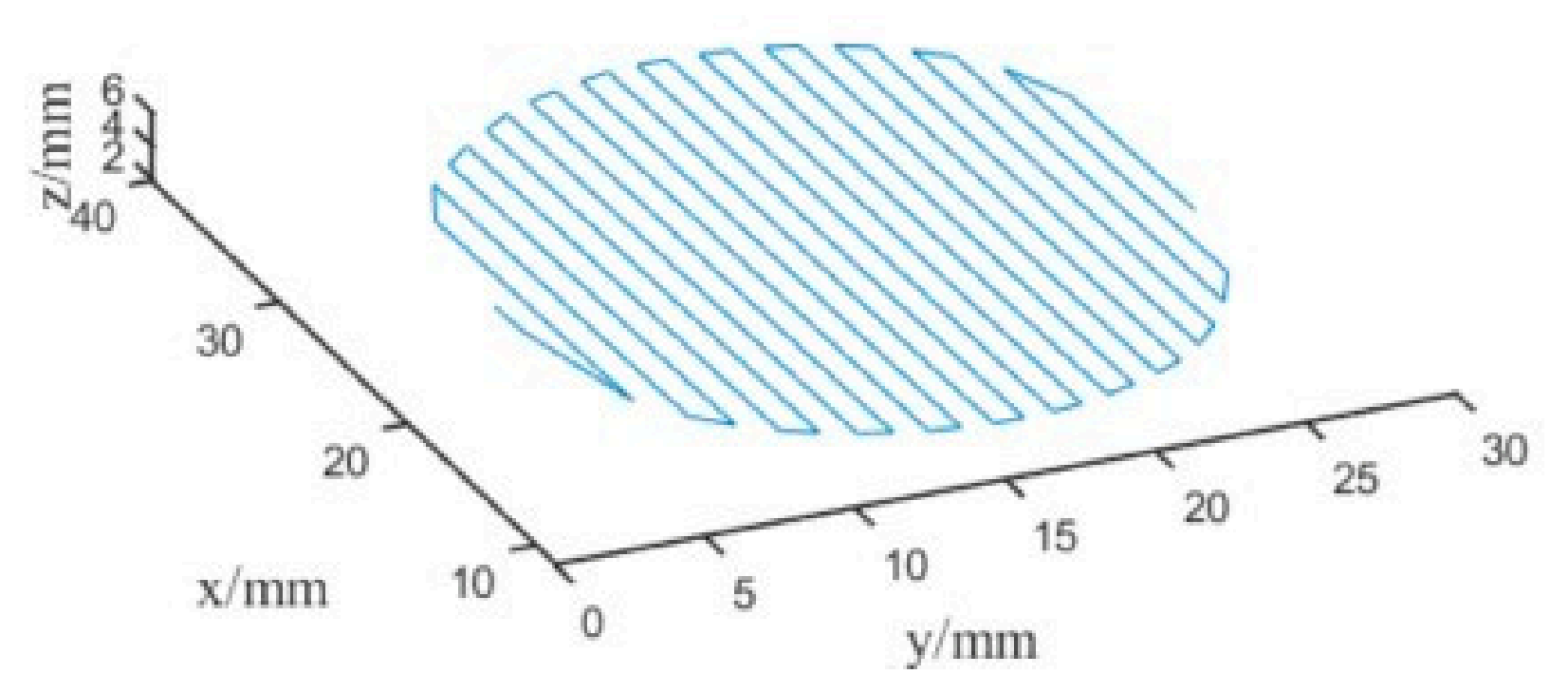
Tool contact path diagram.

Based on the boundary of the area to be processed, the “row cutting method” is used to plan the trajectory of the tool, and the tool contact points are selected according to the angle between the normal vectors of adjacent contact points, making the planned trajectory smoother. Due to the small machining allowance, the principle of uniformity of the allowance is not considered for the time being, and all the allowances are removed at once. The planning process of the tool contact points is as follows:

1. Divide the curve at an appropriate interval with equal parameter intervals u, and record the y-coordinate value corresponding to the starting point of each curve.
2. As shown in Figure 9, generate parallel lines to the x-axis based on the projection points of the starting points of each curve in the xoy plane, and determine whether there are intersection points between each line and the boundary segments obtained in Section 4. If there are no intersection points, remove the corresponding u-direction curve; if there are intersection points, record the minimum and maximum x-axis values ${\;x}_{min\;}$ and ${\;x}_{max\;}$ of the intersection points of each line.
3. On the u-directional curves obtained from step 2, plan the tool positions. Let the total number of tool positions to be planned along the entire u-directional curve be n. The u parameter of each curve is fixed. Starting from v=0, extract the tool positions on the corresponding u-directional curve at the preset parameter intervals (1/n) while checking whether the angle between the unit normal vector of this point and the normal vector of the previous tool contact point exceeds the threshold. If not, this point can be used as the tool contact point. If so, reduce the parameter interval here to 1/2n, reselect the points to calculate the normal vector angle, and continue until an appropriate tool position is obtained. After recording the unit normal vector $e_{1}$ and the tool contact point coordinates $p_{1}$ corresponding to parameter $v_{1}$, set parameter $v_{2}=v_{1}+1/n$, calculate the unit normal vector $e_{2}$ and the coordinate position $p_{2}$ of the next position on the curve, and calculate the angle $\theta_{1}$ between $e_{1}$ and $e_{2}$. If $\theta_{1}$ > $\theta_{thred}$, then discard the calculation result at $v_{2}$, set $v_{2}^{\prime }=v_{1}+1/2n$, and calculate the normal vector angle $e_{2}^{\prime }$ between the new position and $e_{1}$, and the angle $\theta_{1}^{\prime }$ between them. If $\theta_{1}^{\prime }<\theta_{thred}$, then include this position as a tool contact point and select the next tool contact point based on this point. If not, reduce the parameter interval by half and recalculate until the angle is less than the threshold. If $\theta_{1}<\theta_{thred}$, continue to select tool positions at a parameter interval of 1/n and record the normal vectors $e_{i}$ and spatial coordinates $p_{i}$.
4. Based on the individual tool positions extracted from the u-direction curves in step 3, since the angles between the normal vectors of adjacent tool positions all meet the threshold requirements, the trajectory direction will not undergo significant changes. Subsequently, the points outside the boundary curve are removed from all the tool contact points. The x-coordinates $x_{i\;}$ of each tool position on the u-direction curve are compared with the $x_{min\;}$ and $x_{max}$ calculated in step 2 of the corresponding process. Only the tool contact points $A_{k}\ldots A_{m}$ that satisfy $x_{min\;}{<x}_{k},\ldots x_{m}{<x}_{max}$ are retained, as well as the adjacent $A_{k-1}$ and $A_{m+1}$ points, thereby eliminating the tool contact points on the u-direction curve that are outside the processing area boundary line.
5. Repeat the calculations of steps 3 and 4 for the next u-direction curve in sequence. As shown in Figure 10, connect the tool contact points calculated for each u-direction curve in sequence to form an s-shaped tool contact trajectory.
6. On the s-shaped tool contact trajectory, the tool position coordinates $q_{i}$ are calculated successively according to Equation (24). Using the calibration results of the scanner coordinate system and the workpiece coordinate system, each tool position coordinate $q_{i}$ is transformed into the workpiece coordinate system to form an ordered point set $Q_{i}$. During the processing, the center of the ball-end milling cutter passes through $Q_{i}$ successively, which is the processing trajectory planning strategy of this paper.
  (24) $$q_{i}=p_{i}+r\times e_{i},$$
  where $p_{i}$ is the spatial coordinate, $e_{i}$ is the normal vector, and r is the radius of the fitted circle.

## 6. Milling Experiment and Results

### 6.1. Test Platform

**Figure 11:**
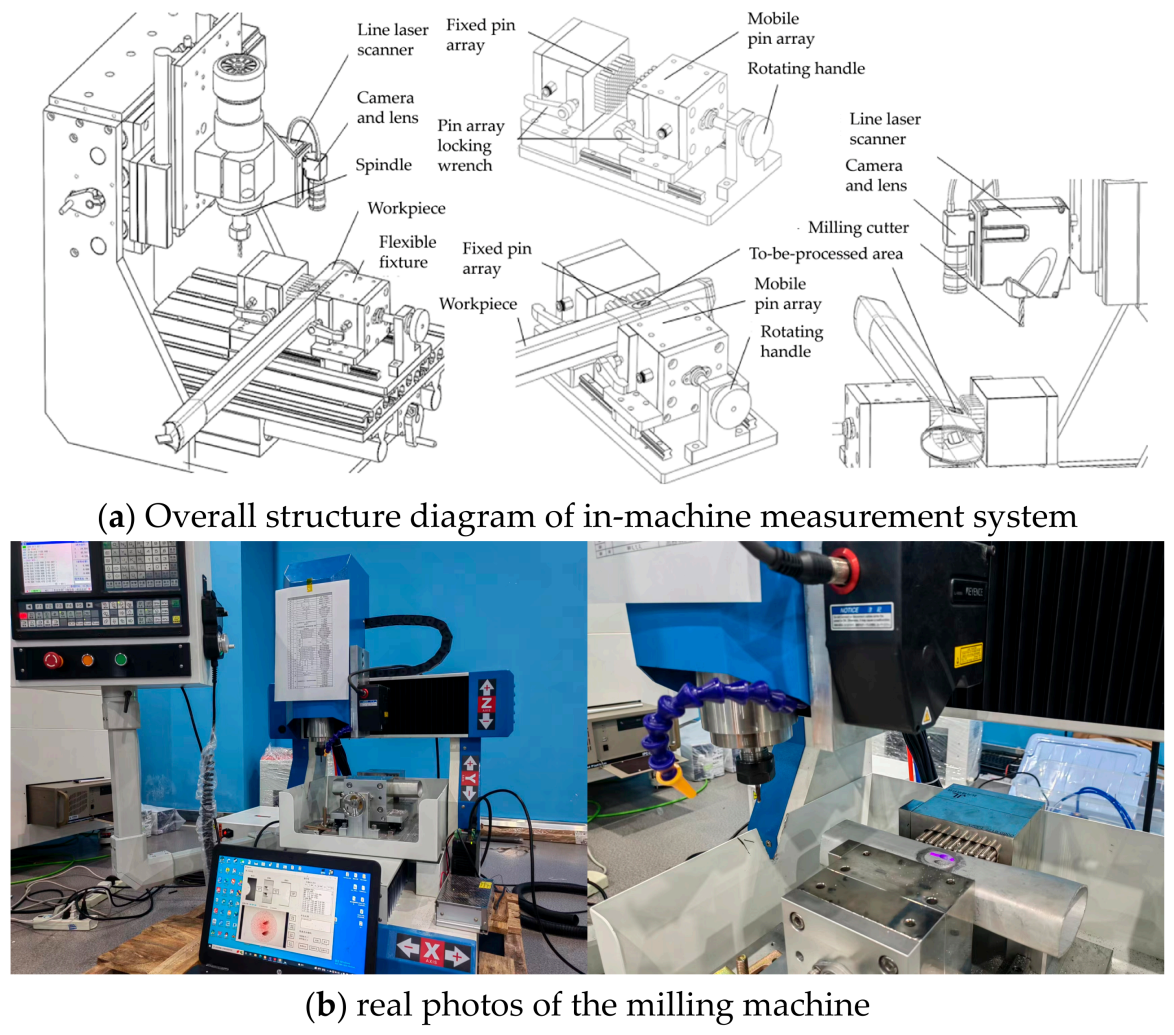
Overall structure diagram of in-machine measurement system and real photos of the milling machine.

The adaptive machining system presented in this paper is built upon a three-axis gantry vertical milling machine, specifically the Model 300C from Dongguan Mikie CNC Technology Co., Ltd. (Dongguan, China), as shown in Figure 11. The flexible fixture is fixed on the worktable of the machine tool and consists of a fixed pin array and a moving pin array that are arranged relative to each other. The moving pin array can move closer to or away from the fixed pin array. Each pin has a spring at one end, which positions the area of the workpiece to be machined between the pin arrays and makes the machined surface as level as possible. Then, the handle is rotated. When the workpiece surface is in full contact with the pin array and forms a conformal support, the pin array is locked to complete the clamping. The industrial camera and line laser scanner are fixed on the *Z*-axis of the milling machine through an installation frame and can move synchronously with the spindle.

### 6.2. Milling Test and Results

First, an industrial camera was used to capture the image of the clamped part at a fixed position above the assembled workpiece. Using the template matching algorithm introduced in Section 3.1, the image coordinates of the center position of the area to be machined were extracted, and the corresponding position of this point on the $O_{W}-X_{W}Y_{W}$ plane of the workpiece coordinate system was calculated based on the calibration results of the industrial camera. This step completed the initial positioning of the area to be machined. To quantitatively evaluate the accuracy of this positioning stage, a high-precision ceramic checkerboard target was used for repeatability verification experiments. Multiple feature points on the target were repeatedly located 10 times under the same working distance, and the standard deviation of the image coordinates was calculated. Experimental results demonstrate that the localization repeatability of the template matching algorithm was better than 2 pixels (with a pixel size of 0.8 μm), which satisfied the initial positioning accuracy requirement for subsequent scanning area planning.

The LJ-X8060 line laser scanner (Keyence Corporation, Osaka, Japan) features a measurement accuracy of 3 μm in depth and 5 μm in the lateral direction, with a repeatability of 0.3 μm for both the Z-axis and X-axis, and operates with a scanning line width of 16 mm. The through-hole aluminum block to be machined and the surrounding welding area were approximately 30 mm long and 22 mm wide. Therefore, the center position of the processing area on the $O_{W}-X_{W}Y_{W}$ plane of the workpiece coordinate system was used as the center of the rectangular scanning area, which was planned to be 45 mm long and 31.5 mm wide. The entire area was divided into two parts with a width of 16 mm along the width direction for scanning, and the adjacent overlapping area width was 0.5 mm. In terms of scanner calibration, a system calibration was carried out using standard blocks and a precision ceramic ball array. A total of 36 control points were arranged within a calibration space of 100 mm × 80 mm × 40 mm. The transformation matrix between the scanner coordinate system and the machine tool coordinate system was obtained through least squares fitting. After calibration, residual analysis showed that the average reprojection error of the calibration points was 0.015 mm. When the calibration was repeated 10 times within a depth range of 40 mm, the standard deviation of the measurement results’ repeatability was less than 0.003 mm, indicating that the calibration process has high stability and repeatability.

The scanned point cloud data were sequentially processed through filtering and denoising, registration and fusion, extraction of the pipe-shell point cloud, boundary fitting of the region of interest, and NURBS surface construction. For boundary extraction, a region-growing algorithm based on normal-vector differences was adopted, which demonstrated good robustness under varying operating conditions. After point cloud segmentation, the extracted tube shell point cloud served as the fitting data for subsequent surface reconstruction. To exclude the possibility that this portion of the point cloud could inadvertently include points from the machining region, a quantitative analysis was conducted as follows. Since there exists a distinct height transition between the machining region and the tube shell surface, the presence of anomalous points in the extracted boundary can be identified by examining the height differences between adjacent points in the segmented tube shell point cloud. The boundary extraction procedure was applied to 20 randomly selected workpieces. For each workpiece, a narrow annular band approximately 4 mm in width, extending outward from the extracted internal boundary into the tube shell region, was defined as the analysis area. The analysis shows that 95 percent of the Z-direction height differences between adjacent points were below 0.010 mm, and the maximum observed difference was less than 0.015 mm. Given the extremely small and consistent height differences between adjacent points, it can be concluded that the extracted point cloud boundary did not include points from the machining region, thereby ensuring that the fitting points used for subsequent surface reconstruction were all derived from the tube shell surface. For the NURBS surface reconstruction, the surface order was chosen as 3 (*p* = 3, *q* = 3), the control-point count was adaptively determined according to the partitioned point cloud data, the node vector was obtained using the Hartley–Judd scheme, and the smoothing weight was set to 0.1. The fitting quality of the surface reconstruction was quantitatively evaluated using the distance from points to the surface. A total of 500 points were randomly sampled on the reconstructed surface, and the distance between their corresponding points and the original point cloud was calculated, resulting in a root mean square error of 0.023 mm and a maximum deviation of 0.061 mm, indicating that the constructed NURBS surface could accurately reflect the morphological features of the area to be processed.

Based on the appropriate feed rate, the line-cutting strategy was adopted for extracting the tool contact points and calculating the tool position points. A ball-end milling cutter with a diameter of 6 mm was employed, with a feed rate of 600 mm/min and a depth of cut of approximately 0.31 mm. The processing times reported here represent average values calculated from the 20 independent experiments. Prior to machining, line laser scanning was performed according to the planned scanning area. Line laser scanning, including visual positioning and data acquisition, took approximately 50 s per workpiece. Following data acquisition, point cloud stitching, filtering, NURBS surface reconstruction, and tool path generation were completed in approximately 70 s using a standard industrial computer equipped with an Intel Core i7-1360P at 2.20 GHz and 16 GB RAM. The subsequent milling operation took approximately 240 s per workpiece. The total cycle time per part was consistently maintained under 6 min.

**Figure 12:**
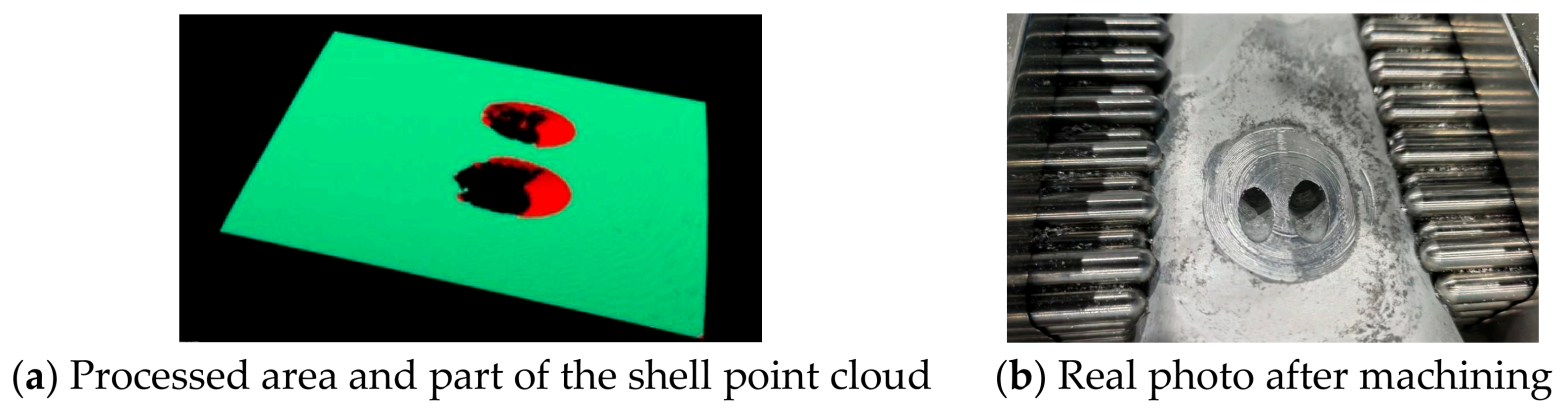
Processed area and part of the shell point cloud and a real photo after machining (green: machined region; red: original hole region).

The tubular shell workpieces were machined on a CNC machine tool using the generated NC codes. Upon completion of the machining process, the finished surface was scanned along the same scanning path as that employed for acquiring the raw blank morphology. The acquired point cloud data were then processed using a region-growing segmentation algorithm to differentiate the curvature characteristics across various regions of the finished surface, with the results presented in Figure 12. From the point cloud data, it is evident that the vast majority of the machining allowance in the processed region were removed after milling. The locally rough areas on the finished surface predominantly appeared on the unmachined portions of the tubular shell, indicating that the surface smoothness of the regions milled using the proposed method is comparable to that of the unmachined shell areas. Furthermore, a smooth surface finish was observed at the transitional boundary between the machined and unmachined regions.

**Figure 13:**
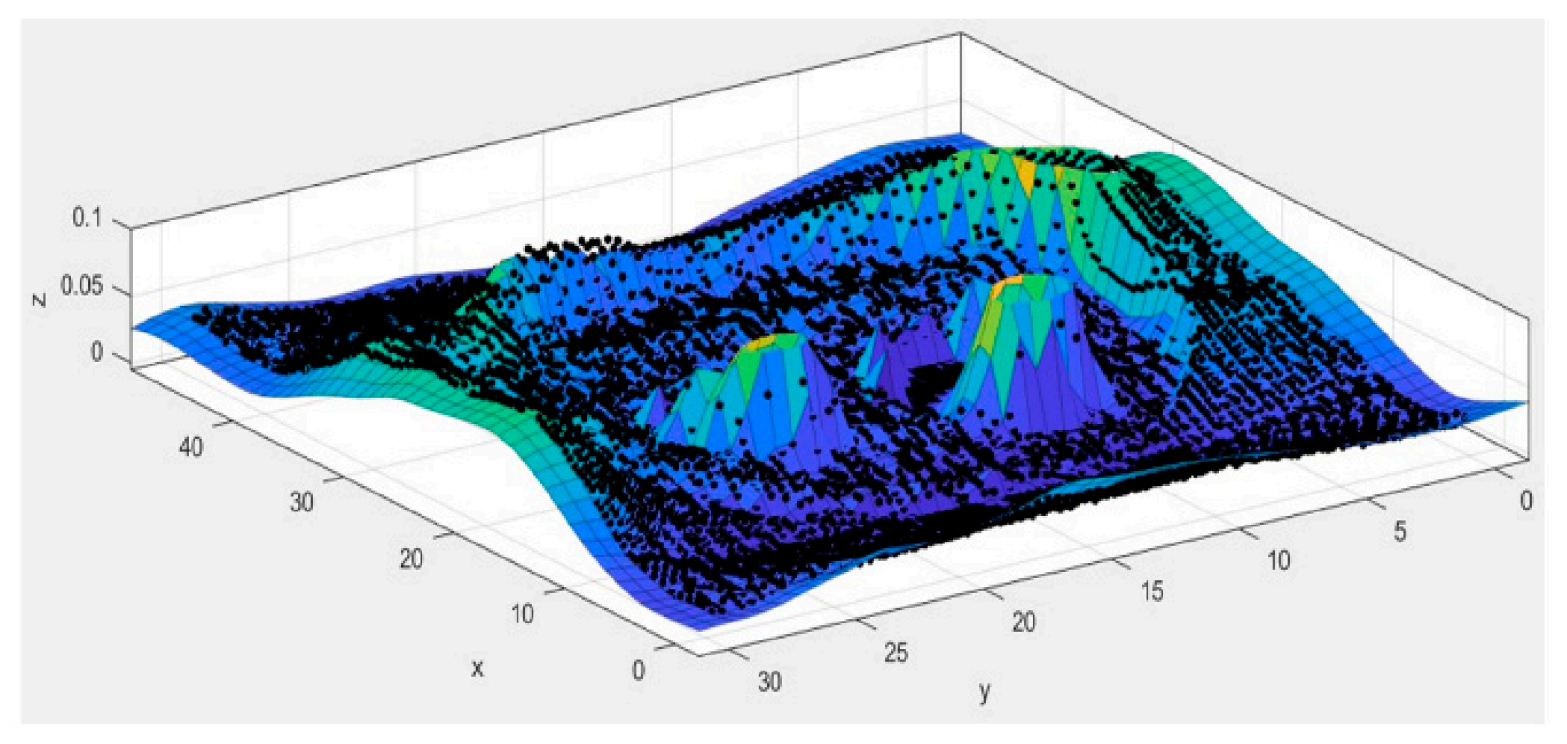
Machining error distribution diagram. The color map indicates error magnitude (cool = small, warm = large, darker = smaller); black points denote the error values.

The distance from the intersection point between the normal vector of a certain point on the finished product surface and the theoretical surface after processing to that point is taken as the processing error to evaluate the processing accuracy. The calculated error distribution is shown in Figure 13. Due to the presence of noise points at the hole locations in the central region of the workpiece, the error calculation results in this area were excluded from the statistical analysis. Quantitative analysis was performed on a total of 1200 measurement points, with 60 points sampled from each of the 20 independently machined workpieces. The overall mean machining error across all points was 0.042 mm, with a standard deviation of 0.023 mm. The mean errors for individual workpieces varied from 0.035 mm to 0.051 mm, and the standard deviation of these 20 means was 0.005 mm, indicating good process repeatability. The error distribution exhibited a median of 0.039 mm and a 90th percentile of 0.072 mm. The cumulative distribution function of the errors, plotted in Figure 13, shows that 77.1 percent of the points fell below the 0.055 mm threshold. A 95 percent confidence interval for the mean error was calculated from 0.039 mm to 0.045 mm, confirming that the process reliably met the required accuracy specifications. For this type of adaptive finishing operation, the industry acceptance criterion is generally specified as a maximum machining error of less than 0.1 mm. The maximum machining error observed across all experiments remained consistently below this 0.1 mm threshold, demonstrating that the proposed method fully satisfied the industrial requirement. The standard deviation of 0.023 mm for individual measurement points and the standard deviation of 0.005 mm for the mean errors across the 20 workpieces collectively confirm that the observed experimental variability was well within acceptable limits and did not compromise the reliability of the proposed method. In summary, the adaptive machining scheme proposed in this paper can effectively remove most of the machining allowance on the protruding areas of workpieces, significantly improve the processing efficiency of this operation, and provide a favorable process foundation for subsequent polishing.

## 7. Conclusions

1. This paper investigates key technologies of adaptive machining within a proposed in-machine measurement system. The system encompasses calibration of the measurement setup, automatic localization of the processing area, acquisition and processing of measurement data, and machining trajectory planning. Under conditions of under-positioning and workpiece clamping, the system enables complete acquisition of measurement information, allowing for adaptive adjustment of the finished surface according to the blank morphology. Experimental results indicate that 77.1 percent of the machining errors are below 0.055 mm, with a maximum error less than 0.1 mm, meeting the industry requirement for adaptive machining.
2. To address the challenges posed by workpiece under-positioning and variations in the processing area, a multi-sensor detection strategy is adopted, where an industrial camera provides initial positioning, followed by precise line laser scanning. This approach reduces ineffective scanning travel and enhances the automation level of the overall system. The repeatability and robustness of the proposed methodology are validated through 20 independent repeated experiments across the complete process chain.
3. The proposed line laser scanning point cloud stitching method, which leverages the feed and positioning functions of the CNC machine, effectively mitigates the trade-off between scanning line width and scanning accuracy. This improves the efficiency of acquiring high-precision, complete point cloud data for the measured object within the in-machine measurement system. Practical limitations of the method include degraded performance on highly specular surfaces and reliance on sufficient visual features for reliable boundary extraction. Overall, the proposed adaptive machining scheme effectively removes the majority of machining allowance from protruding regions, improves processing efficiency, and provides a favorable surface condition for subsequent polishing.
4. Despite the promising results demonstrated in this work, several practical limitations must be acknowledged to guide future deployment in industrial settings. A quantitative decomposition of the overall machining uncertainty into individual processing stages is not established in the current study, although the accuracy of key intermediate steps, including boundary extraction and NURBS surface reconstruction, are individually validated in Section 6.2. Without such a systematic uncertainty budget, the relative contribution of each stage to the final machining error remains unquantified, which represents an important gap for process optimization. The current system exhibits reduced robustness under varying illumination, susceptibility to surface contamination and oxide layers, and degraded scanning quality on highly reflective aluminum surfaces due to specular interference. Furthermore, the experimental validation is confined to workpieces within a limited size range, and the applicability to large-scale components remains unverified. Extension to five-axis machining systems would also necessitate additional calibration and kinematic modeling. To overcome these challenges, future work will focus on the following directions: (a) developing adaptive exposure control and multi-directional scanning strategies to enhance robustness against varying illumination and reflective surfaces, (b) investigating partitioned scanning combined with global registration methods for large-scale component inspection, (c) extending the current framework to five-axis systems through systematic kinematic calibration, and (d) establishing a comprehensive quantitative uncertainty budget that rigorously decomposes and quantifies the contribution of each processing stage, thereby providing a more definitive basis for prioritizing algorithm improvements. Addressing these aspects will further enhance the versatility and industrial viability of the proposed adaptive machining system.

## Data Availability

The original contributions presented in this study are included in the article. Further inquiries can be directed to the corresponding author.
